# Supplementary material for: Associations of Motor Performance and Executive Functions: Comparing Children with Down Syndrome to Chronological and Mental Age-Matched Controls
Source: Children (Basel). 2022 Jan 5;9(1):73. doi: 10.3390/children9010073 (PMC8774164; doi:10.3390/children9010073)
Supplement: Supplementary file 1 [file children-09-00073-s001.zip › children-1495222-supplementary.pdf]

Table S1. Correlation matrix of participants' motor performance (percentiles), demographic characteristics, body composition and IQ.

MABC-2				
	**Manual dexterity**	**Ball skill**	**Postural Control**	**Total Test Score (TTS)**
**Down - Syndrome**				
**Age**	-.522	.159	-.406	-.183
**Sex**	.000	-.115	.164	.192
**BMI percentile**	-.610*	-.466	-.612*	-.534
**Palm-to-finger- length ratio**	.214	-.333	.219	-.017
**Sports participation**	-.170	-.122	-.254	-.028
**PPVT IQ**	-.348	.535	-.249	-.035
**CA-adjusted**				
**Age**	-.784**	.533	-.024	-.162
**Sex**	.114	-.446	.431	-.169
**BMI percentile**	.217	-.588*	.292	-.580*
**Palm-to-finger- length ratio**	.110	-.117	-.160	-.079
**Sports participation**	-.071	-.055	-.155	-.413
**PPVT IQ**	.280	-.144	-.079	.056
**MA-adjusted**				
**Age**	.100	-.231	-.168	-.157
**Sex**	.252	.577*	.623*	.527
**BMI percentile**	.231	-.436	-.218	-.132
**Palm-to-finger- length ratio**	-.075	-.244	-.150	-.065
**Sports participation**	.103	-.376	-.021	-.078
**PPVT IQ**	.173	.183	.255	.271

Note. * $p < .05$; ** $p < .01$; CA = chronological age; MA = mental age.
